# Warming-Induced Decline of *Picea crassifolia* Growth in the Qilian Mountains in Recent Decades

**DOI:** 10.1371/journal.pone.0129959

**Published:** 2015-06-29

**Authors:** Li Yu, Lei Huang, Xuemei Shao, Fengjing Xiao, Martin Wilmking, Yongxiang Zhang

**Affiliations:** 1 National Climate Center, China Meteorological Administration, Beijing, 100081, China; 2 Institute of Geographic Sciences and Natural Resources Research, Chinese Academy of Sciences, Beijing, 100101, China; 3 Ecosystem Dynamics, Institute for Botany and Landscape Ecology, University Greifswald, Grimmer Strasse 88, 17487, Greifswald, Germany; Chinese Academy of Sciences, CHINA

## Abstract

Warming-induced drought has widely affected forest dynamics in most places of the northern hemisphere. In this study, we assessed how climate warming has affected *Picea crassifolia* (Qinghai spruce) forests using tree growth-climate relationships and the normalized difference vegetation index (NDVI) along the Qilian Mountains, northeastern Tibet Plateau (the main range of *Picea crassifolia*). Based on the analysis on trees radial growth data from the upper tree line and the regional NDVI data, we identified a pervasive growth decline in recent decades, most likely caused by warming-induced droughts. The drought stress on *Picea crassifolia* radial growth were expanding from northeast to southwest and the favorable moisture conditions for tree growth were retreating along the identical direction in the study area over the last half century. Compared to the historical drought stress on tree radial growth in the 1920s, recent warming-induced droughts display a longer-lasting stress with a broader spatial distribution on regional forest growth. If the recent warming continues without the effective moisture increasing, then a notable challenge is developed for *Picea crassifolia* in the Qilian Mountains. Elaborate forest management is necessary to counteract the future risk of climate change effects in this region.

## Introduction

The rapid warming over the last half century is unequivocal, and many observed changes are unprecedented. More than half of the observed increase in global average temperature is caused by anthropogenic forcing [1]. This anthropogenic warming affects all ecosystems, notably those at high latitudes and in alpine regions [2–4]. This warming not only causes temperature limitations for certain plant species [5] but also induces consequent droughts because of the changing hydrothermal conditions at the regional scale [6]. According to recent research, regional droughts in certain areas are intensifying and will become more frequent in the future as a result of recent warming [7].

As the dominant ecosystem in many mountain areas, forests are expected to experience some of the most dramatic warming [8] and show consequent responses to warming induced drought in areas of the northern hemisphere [5, 9]. The significant declines in forest dynamics are caused by a widespread moisture-driven drought in the tropical forests in the Amazon basin [10, 11], temperate forests in the western United States [12], and trembling aspen stands in western Canada [12–14]. This large distribution of forest decline may cause a significant change in the terrestrial carbon sink [15–18]. Therefore, forest dynamics and the response of trees to recent warming must be investigated, notably in the mid-latitudes of the northern Hemisphere.

On the Tibet Plateau (TP), which is called the “third pole of the earth”, an observed water deficit appeared along the periphery, notably in the northern and northeastern portion [19], and pronounced warming has occurred in recent decades [20]. Forests on the TP play an important role in regulating water flow of those rivers which rise from the TP; these rivers are crucial for local agriculture and ecology. Because of the large distribution and old ages, the dominant coniferous species on the TP, *Picea crassifolia*, has been widely used in dendroclimatological and dendroeccological research [21–24]. Generally, dendrochronologists mainly investigate the signal that has been recorded in trees and how to extract the signal for paleo-reconstructions. Only a few studies focus on how varying regional hydrothermal conditions effect the growth of *Picea crassifolia* and forest dynamics at spatial scales during the recent warming [25]. The general hypothesis on tree growth-climate relationship is that trees growing at upper elevation tree lines are more sensitive to temperature, and trees growing lower than this position are moisture limited [18, 26]. Prior studies found that Picea crassifolia at its upper tree line in Qilian Mountains are limited mainly by temperature and/or moisture and trees growing lower than upper tree line are mainly limited by moisture [27, 28]. Therefore, investigating the forest dynamics and tree growth-climate relationship from tree lines could generally reflect how this warming affects the entire forest dynamics and tree growth. The objectives of this study were to explore the effect of varying regional hydrothermal conditions on the radial growth and dynamics of *Picea crassifolia*, one of the dominant coniferous species on the TP. The regional hydrothermal conditions reflected recent warming over a large portion of the natural climatic envelope of *Picea crassifolia*. Therefore, this study was designed to elucidate the spatial-temporal variability of recent *Picea crassifolia* growth and regional forest dynamics variation over the last century.

## Materials and Methods

### Tree ring data

**Fig 1:**
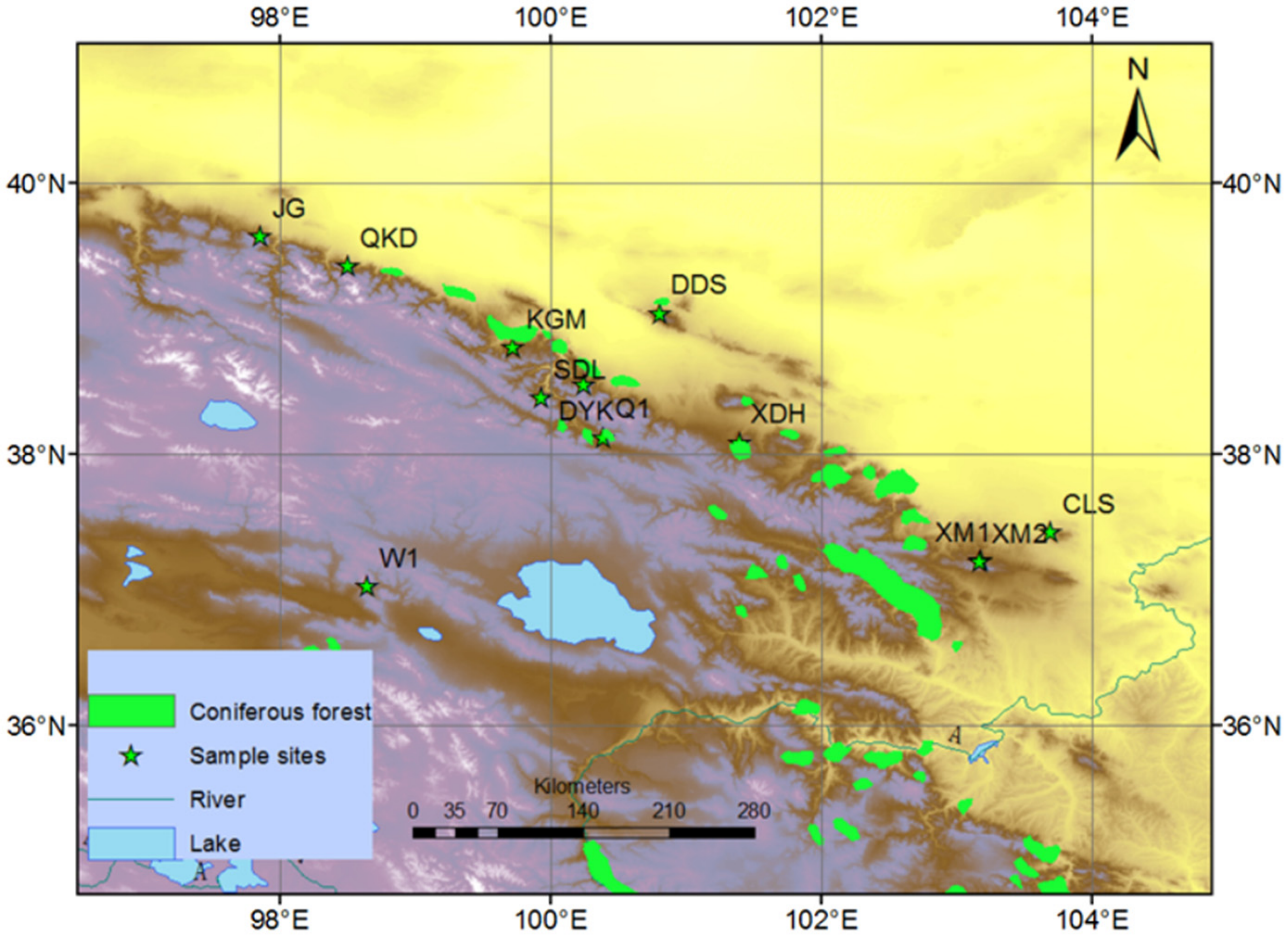
Study area and sample sites. Green stars indicate samples sites, black crosses indicate the gridded data for CRU and green the shaded area indicate the distribution of the coniferous forest in situ.

*Picea crassifolia* is a shade tolerating species growing at locations with annual precipitation of approximately 400–700 mm. In this study, trees from 12 sites were sampled from upper or close to upper tree lines in Northeastern TP (Fig 1). All field and sampling work have been done with the permission from the forestry bureau of Wulan. All series of increment cores of each site were taken from dominant and co-dominant trees which appeared healthy, were relatively isolated, and were close to their upper limit (Table 1). In total, 317 trees were collected in this study. Site elevations display the approximate topography of these mountain chains. All cores were processed by following standardized dendrochronological methods [26]. Referring to prior research [25], RES chronologies were employed to investigate the spatial and dynamic effect of the regional hydrothermal condition on *Picea crassifolia* growth at the upper/near forest line (S1 Table). To evaluate the shared variance of the chronologies network, a principal component analysis (PCA) was performed based on the correlation matrix during the common period (1900–2005).

**Table 1:** Characteristics of the sample sites and site chronologies.

Site	Latitude(N)	Longitude(E)	Elevation	Sample size(tree/cores)	Length of chronology
**W1**	37.03	98.65	3777	11(22)	1690–2005
**Q1**	38.13	100.39	3357	23(41)	1642–2005
**SDL**	38.42	99.94	3304	30(30)	1785–2005
**DDS**	39.04	100.81	2826	28(33)	1779–2005
**XM1**	37.22	103.18	3000	26(41)	1838–2005
**XM2**	37.21	103.17	3000	18(19)	1845–2005
**KGM**	38.79	99.73	2900	24(29)	1848–2005
**DYK**	38.52	100.25	3040	24(32)	1780–2005
**XDH**	38.09	101.40	2755	22(40)	1770–2005
**QKD**	39.39	98.51	2800	20(33)	1850–2005
**JG**	39.61	97.86	2852	15(31)	1727–2005
**CLS**	37.43	103.70	2447	20(20)	1852–2005

### Changes in forest dynamics

Basal area increment time-series consist of a long-term positive trend and with a level off or declines when trees encounter stress or enter senescence [29]. In addition to ring width chronologies, the basal area increments (BAI) were used to reconstruct historical episodes of vegetation dynamics declines and releases. The BAI were calculated using Eq (1):

$$BAI=\pi R_{t}^{2}-\pi R_{t-1}^{2}$$ (1)

where Rt and Rt-1 are the stem radial increments at the end and the beginning of a given annual ring increment, respectively. The site BAI series is calculated based on the mean R of trees from the identical site.

To remove the age effect, BAI series were detrended by a power transformation and then normalized [30]. A sequential application of Student’s t test for change-point detection was applied on the normalized series [31]. Ten years was selected for the cut-off length of the determined growth phase. We ran the analysis on all site-level BAI time-series. The change-point locations for each year have been collected during the period covered by the tree-ring data. A Bayesian calculation with an uninformative prior distribution was employed to check the confidence level of the change-point [32]. The positively (or negatively) identified change-point represents the start of growth decline (or increase). Typical episodes of forest dynamics variation were determined by two reversing changing points. To obtain regional trend results, the mean of all standardized site BAI (SPI) was detected by using the above mentioned methods.

### NDVI data

The normalized difference vegetation index (NDVI), a satellite measurement of surface greenness, is an effective way to represent the vigor of forest in summer. The GIMMS NDVI dataset with 8 km resolution from 1982–2006 was used in this study [33]. The GIMMS NDVI dataset is a vegetation index product developed by NASA GSFC (Goddard Space Flight Center) GIMMS (Global Inventor Modeling and Mapping Studies) group, which is synthesized over a 15 day (15 d) period at 8 km resolution. The GIMMS NDVI dataset ensures high data quality because it eliminates the effects of volcanic eruptions, solar elevation angles and sensor sensitivity changes with time. Therefore, this dataset has been widely used in global and regional vegetation monitoring [34]. To monitor the dynamics of *Picea crassifolia* forests during recent decades, the pixels of conifer forest NDVI were extracted according to the distribution within our study area. The yearly change of each pixel was calculated. Trend curve models were employed to predict a change trend by regression analysis. The change trend of the NDVI of each pixel was modelled using 24 years of data during 1982 to 2005, which represents the inter-annual change of forest covered area. The equation is expressed as the following:

$$slope=\;\frac{n\times \sum_{k=1}^{n}k\times Y_{NDVIk}-\sum_{k=1}^{n}k\sum_{k=1}^{n}Y_{NDVIk}}{n\times \sum_{k=1}^{n}k^{2}-{\left(\sum_{k=1}^{n}k\right)}^{2}}$$ (2)

where k ranges from 1 to n, n stands for the year number, and YNDVIk indicates the average NDVI value of vegetation during the growing season of the k^th^ year. The inter-annual NDVI change trend of the study area is shown in the change trend image. The trend curve of each pixel indicates the total change trend by regression analysis to the average NDVI series of the growing season. The slope means the slope of this trend curve. If slope>0, it means that NDVI value is increasing, otherwise decreasing.

### Climate data

Concerning the distribution and data quality, the CRU gridded data were employed in the climate analysis. All grid points within N 35°- 42° / E 95°-105° were selected. Clear hydrothermal gradients were noted over our study area. The precipitation decreases from greater than 400 mm/y in the southeast to less than 100 mm/y in the northwest along the mountain chains, whereas the temperature increases from the TP to the peripheral area. The observed precipitation shows an insignificant increasing trend on the central TP and decreasing trends along the TP periphery, whereas evaporation shows an overall increasing trend [19]. Individual meteorological records show that the mean annual temperatures displayed a significantly abrupt increase starting around 1987–1997 [25].

To better reflect the effect of regional hydrothermal conditions on radial tree growth, we employed a climate index (CI) which has been used in other studies [25, 35]. This CI, compared to a PDSI, is ecologically more appropriate to test for the influence of the combination of precipitation and temperature (hydrothermal conditions) on radial tree growth. Higher correlations between tree growth and CI indicate a higher moisture deficit caused by warming induced drought.

### The response of tree growth to climate

The response of single sites to climate change was estimated using correlation relationships. The dynamic spatial effects of regional hydrothermal conditions on the growth of *Picea crassifolia* forests were accessed by calculating moving correlation relationships between PC1 and PC2 of all sites and the regional hydrothermal condition (CI) with a 30 year window over time. To concisely display the results, three time slices were extracted to represent the dynamic response of tree growth to the regional hydrothermal condition. The first time slice consists of the first 30 years of the respective climate record and the corresponding tree growth. The second time slice starts 10 years later. The third time slice covers the last 30 years of the climate record and the corresponding tree growth.

## Results

### Characteristics of chronologies and multivariate analysis

**Fig 2:**
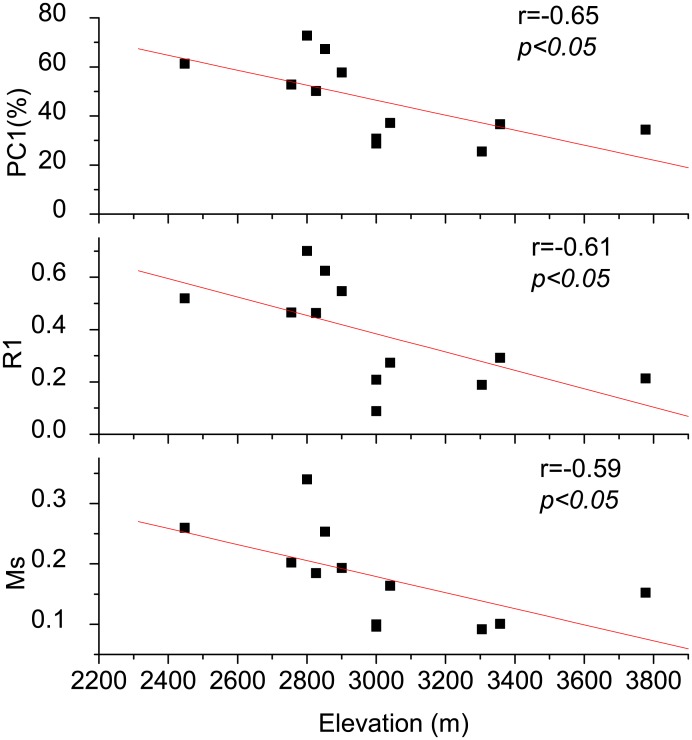
Statistical characteristics of regional tree growth along elevation gradients. Ms is the mean sensitivity which indicates the sensitivity of tree growth to common environmental changes. R1 is the correlation between trees, and PC1 is the population signal of single sites.

The main statistical properties of chronologies display a clear relationship along elevations. Trees from low elevations have higher mean sensitivities (MS). Additionally, low elevations have a higher correlation between trees (R1) and the population signal of single sites (PC1) than trees from high elevations, indicating that trees distributed at low elevations shared more common information than trees distributed at higher elevations (Table 1, Fig 2). The elevation difference between sample sites in this research is larger than 1000 meters. The age span of the forests shared a similar spatial distribution. The oldest trees, approximately 360 years old, grew in the middle of the Qilian Mountains. Younger trees were found at the western and eastern edge of the study area. The first two components explained 38% and 18% of the variance individually and 56% of the total variance cumulatively. A positive loading of all sites for PC1 indicates that all forest sites were regionally affected by a common environmental variable.

### Dynamic relationships between tree growth and regional hydrothermal conditions during the last half century

**Fig 3:**
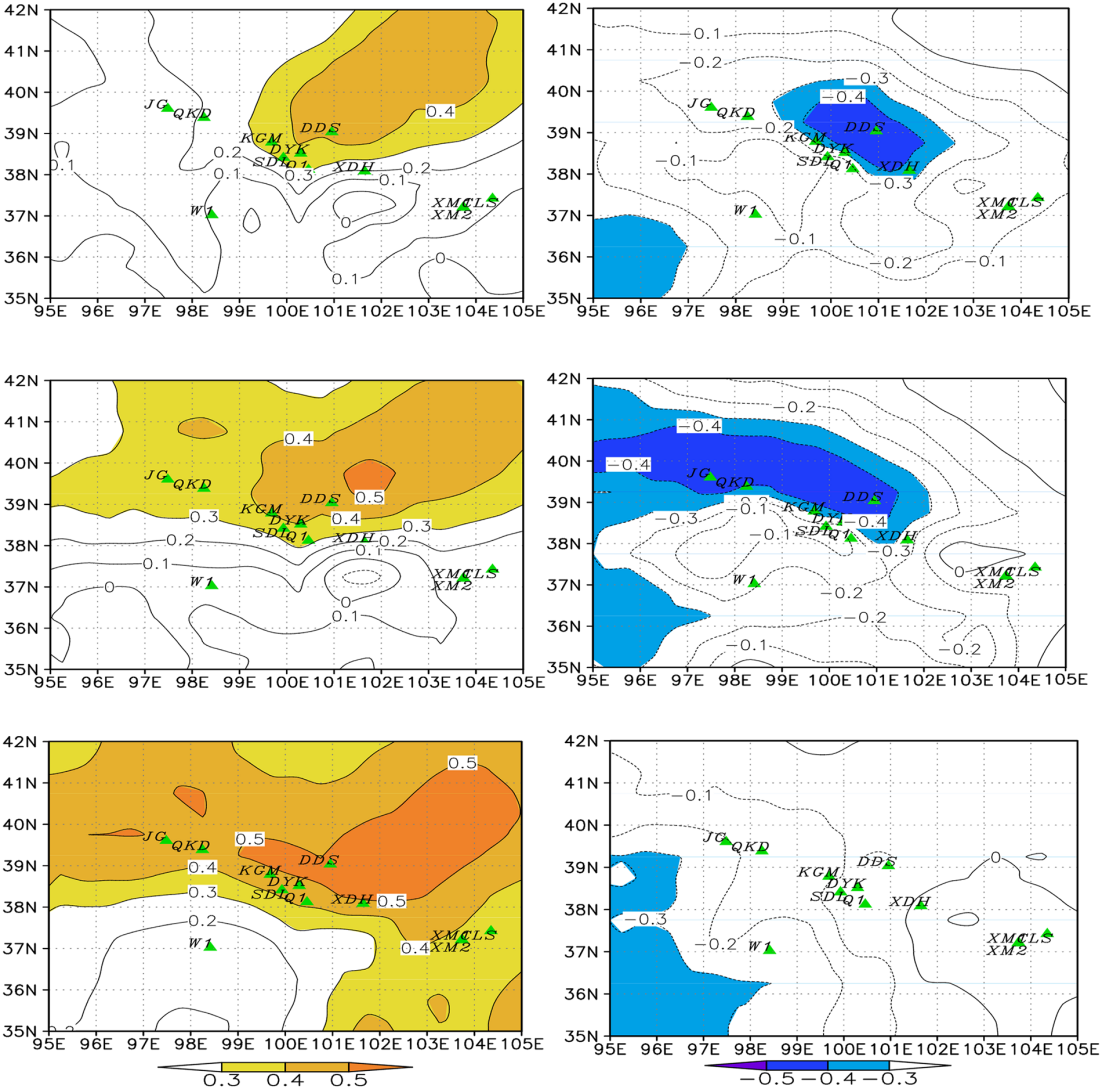
Relationships between tree growth and regional hydrothermal conditions. Spatial correlations between PC1 and PC2 with the gridded climate index (CI). Correlations of PC1 (left) and PC2 (right) with regional CI show the increasing influence of drought stress on our tree ring network over the last half century, concurrent with a retreat of conditions favorable to tree growth. The calculation periods were for (a) and (b) 1951–1982; (c) and (d) 1962–1993; and (e) and (f) 1974–2005. Colored areas are significant at the *a* = 0.1 level.

The single site tree growth-climate relationship shows that trees from most sites have a significant negative correlation with the growing season temperature but lack a significant correlation with precipitation. The dynamic spatial relationship between tree growth and regional hydrothermal conditions shows that PC1 (explains 38%) had a significant positive and PC2 (explains 18%) had a significant negative correlation relationship with CI. These two correlation relationships displayed a clear spatial dynamic variation over time. The positive relationship (drought stress) is expanding in the northeast to the southwest, and the negative relationship (favorable growth conditions) is retreating along the identical direction over time (Fig 3).

### The variation of radial growth and forest dynamics

**Fig 4:**
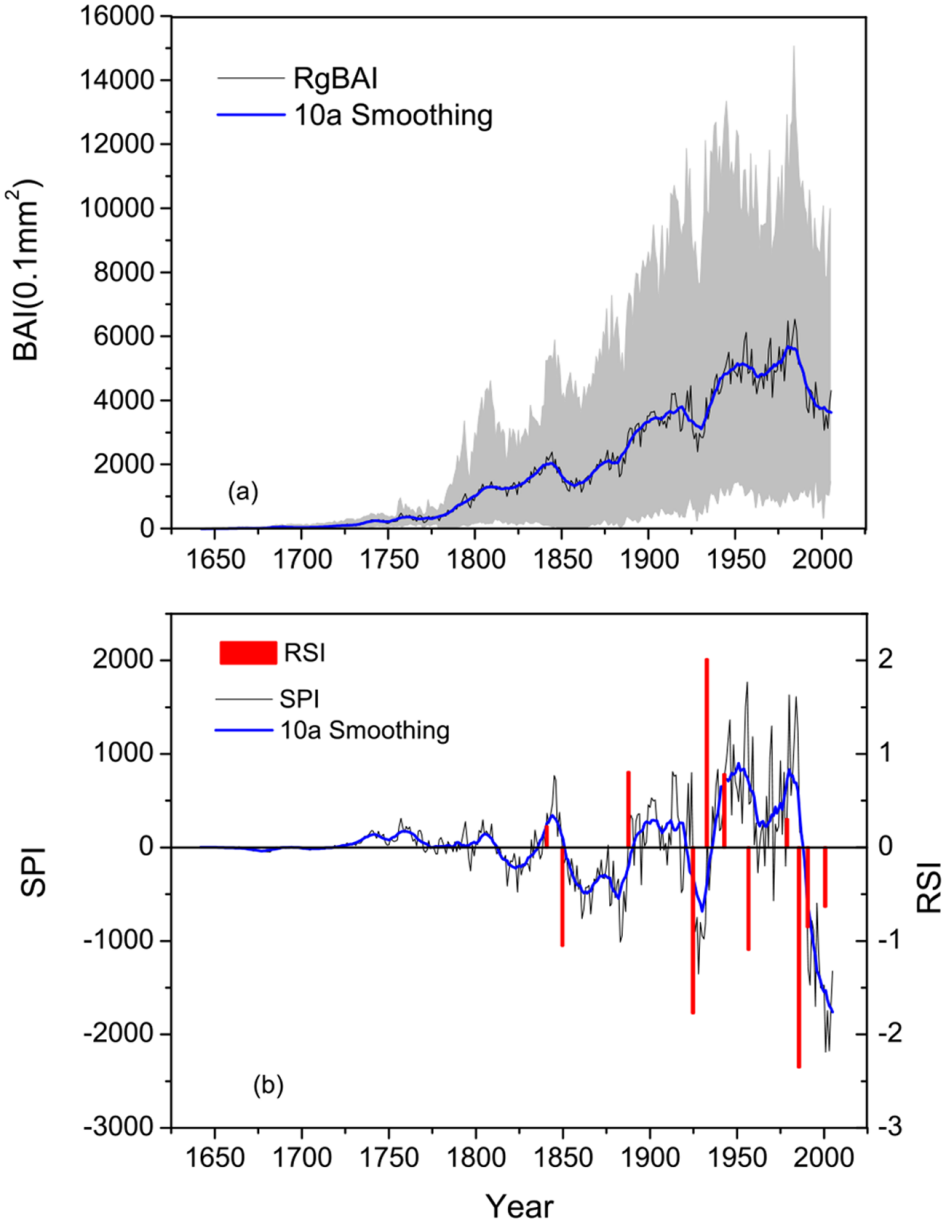
Mean annual basal area increment (BAI) of *Picea crassifolia* (a), and the regime shifts of BAI chronologies (b). In (a), the grey area is the range of the BAI of all sites, the dark curve is the mean annual basal area increment with its 10 year moving average (blue). In (b), the dark curve is the regional BAI chronology (SPI) with its 10 year moving average (blue); the red bar indicates a significant growth trend shift of the regime shift index (RSI).

Regional forest BAI shows a general increasing trend with variation and a notable growth decline in late 20^th^ century. After removing the growth trend, seven typical episodes of forest dynamics variation were identified in the SPI during the last two centuries (Fig 4). The lower growing periods are 1850–1880, 1925–1933, 1957–1979, and 1986–2005. The higher growing periods are 1841–1850, 1880–1925, 1933–1957, and 1979–1986. In terms of the individual sites, differences in the different regimes were noted, but the most common periods are the late 1920s and late 1980s. Relative to the long term mean growth rates over the last century, two notable growth declines were observed in 1920–1940 and after the 1980s at most sites. By contrast, increasing tree growth rates were recently found only at sites W1 and XM1.

**Fig 5:**
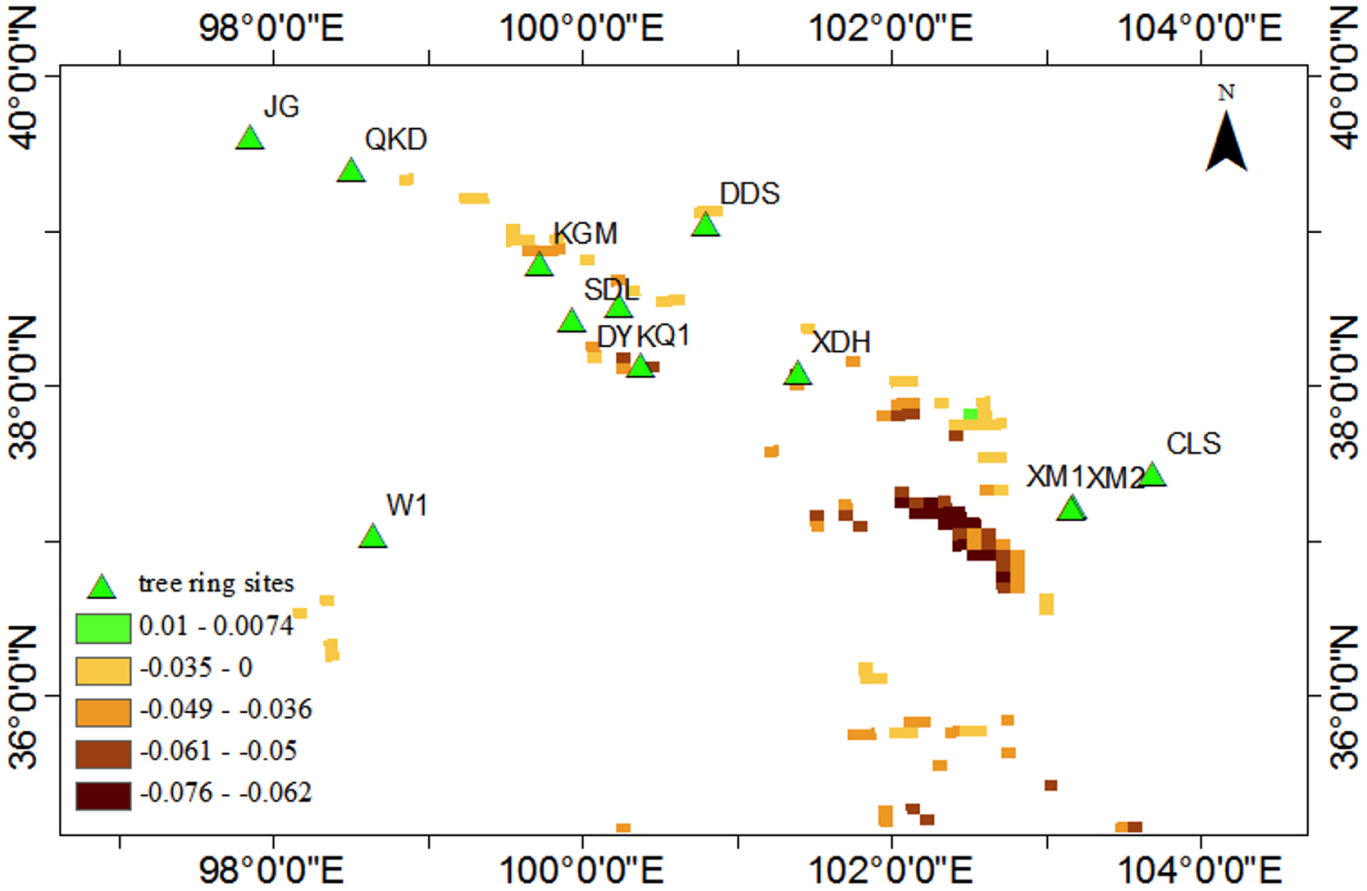
The change of regional conifer forest NDVI. Green triangles are the tree ring sample sites analyzed. The colored squares are pixels for the coniferous forest with 8 km resolution; different levels represent the trend slope of each pixel. The negative slope indicates forest growth decline in each pixel.

*Picea crassifolia* and *Juniperus przewalski* (Qilian Juniper) are two dominant conifer species in our study area. The maximum yearly NDVI of the regional coniferous forest displays a clear decline/browning over last 24 years (Fig 5). A significant decline was noted in all pixels, except for one pixel displaying a slight increasing trend because of problems with technique. The general coniferous growth reduction has spatial differences that show the most of the declines happened in the central eastern part of our study area, and the peripheral area showed a lower decrease.

## Discussion

In this study, we found that *Picea crassifolia* stands and coniferous forests in general were experiencing a significant drought stress. This stress was documented by remote sensing (NDVI) and in situ (BAI) data on the northeastern TP in recent decades. Several similar responses of tree growth-forest dynamics to recent warming have been reported either on a regional or local scale in the Northern Hemisphere in terms of growth decline or even die-off events [2, 36–38]. The rapid warming has also accelerated tree growth decline in semi-arid forests in Inner Asia close to our study area [39]. The water deficit induced by the recent warming is the most likely reason for this wide spread in coniferous forest decline. Based on the experiment of the annual resolution tree ring stable carbon isotope (δ^13^C), [40] illustrated that the warming induced drought stress limited *Picea crassifolia* growth by the iWUE change in recent decades in Qilian mountains. Reflecting on the tree radial growth and forest dynamics, the decline starts slightly after this point and strengthens later. Although studies covering all ecosystems reported that the regional NDVI increased during the last half century over our study area [41], tree ring based regional NDVI reconstruction displayed a decrease in growth during the last 30 years [42]. These divergent trends likely are caused by the different responses of individual ecosystems to recent hydrothermal conditions because regional grassland NDVI increased significantly in the Qilian Mountains [43].

**Fig 6:**
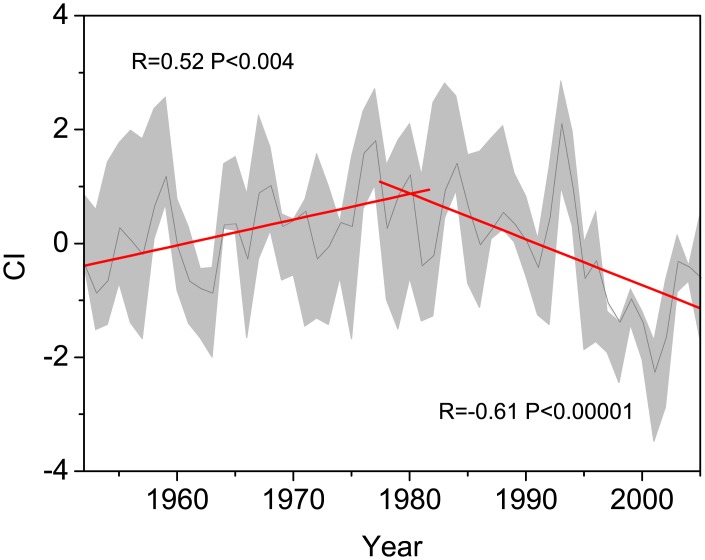
Regional CI variations during 1952–2005 in the study area (N35-42, E 95–105). Grey and red lines indicate regional CI and its 5-year moving average, respectively. The shadow indicates the CI of the 12 sample sites.

Although many studies have predicted that global warming will increase the precipitation in extra-tropical areas, the latest meteorological data indicated that most middle latitude areas will receive less precipitation or more precipitation in extreme rainfall events [1]. According to records from individual meteorological stations in this area, the regional temperature has increased by more than 1.5℃ over the last half century with an abrupt increase after 1980 [44]. Most areas received less precipitation and amplified water limitations because of the increasing warming [19]. The regional CI displayed a significant decrease during 1952–2005 (R = -0.218, P<0.11). Using 1980 as a break point, different trends are noted in the two periods. The first period displayed a significantly increasing CI with R = 0.52 (P<0.003), and the second period displayed a significantly decreasing CI with R = 0.61 (P<0.0001) (Fig 6). The dynamic ecological reaction of *Picea crassifolia* trees show that the drought stress was expanding gradually from the peripheral area of the TP onto the TP, displaying a response to the spatio-temporal variations in the regional hydrothermal conditions (Fig 3). This kind of stress on *Picea crassifolia* radial growth also strengthened along the elevation gradients from upper tree line to lower tree line [28]. Notably, the drought stress may cause not only tree growth decline but also a decrease in the recruitment of trees and an increase in tree mortality and forest die-off. [45] found that the recruitment of *Juniperus przewalskii*, one of the other dominant coniferous species on the Qilian Mountains, decreased after the 1970s at the upper tree line. Tree mortality has been observed in more xeric forest areas in the middle arid Asia neighboring our study area [39]. Droughts caused forest growth decline and is an important climate driver for forest growth in the northeastern TP. During the last century, similar droughts induced declines also occurred in the 1920s. The 1920s drought is the most famous drought during the past few centuries, which caused tree radial growth to widely decline and forest dieback in northwest China [46, 47]. Comparing the two major growth declines during the last century in our study area, we found that tree growth was recorded in an almost similar manner, but with differences in timing and intensity. The 1920s forest reduction was extremely short in time. The 1980s forest growth decline was gradual and was sustained longer.

Upper tree lines are generally assumed to be limited by temperature. Therefore, these lines are the ideal place to investigate the response of forests to the recent warming [48]. Although sampled at the upper tree line, large altitude gradients remained between the sample sites. At the edge of the Qilian Mountains, notably in the peripheral western and eastern area, trees could not grow higher because of topographical (elevation) limitations. Recent dendroclimatological investigations supported that a coherent relationship exists between the tree growth of *Picea crassifolia* and climate factors at the upper tree line or along elevation gradients [22, 49, 50]. Most individual site studies found precipitation holding a dominant effect on tree growth at the beginning of the growing season [51–53]. Select trees growing at higher elevation sites could benefit from this accelerating warming [25]. The rest of the forest, around and below 3700 m, was gradually affected by moisture stress since the 1980s [28]. Both the NDVI and tree growth-climate relationships further indicated that almost the entire spruce forest, not only the upper tree lines, experienced a strengthening drought stress at the northeastern TP. If this warming continues without an effective precipitation increase, then predicting how this species will develop under current climate conditions in our study area is difficult without the knowledge of their water use efficiency and other responses to moisture deficits [54]. Expected future changes in the growth rate of *Picea crassifolia* trees must be considered in forest management decisions. We highly recommend that the knowledge of climate—growth relationships, as represented here, will be combined with adaptive management to reduce the risks and uncertainties associated with forest management decisions.

## Supporting Information

S1 Table RES chronologies from 12 sites we have been used in this analysis. The column name is the site name. Time spans 1900–2005.
(XLSX)
